# A herbivore-inducible CYP82 catalyzes DMNT formation in *Idesia polycarpa* leaves

**DOI:** 10.1186/s12870-026-09677-2

**Published:** 2026-08-20

**Authors:** Melina Panagoulias, Jannik Bäsmann, Stina Strassburg, Maya Bach, Miriam Fernández-Giro Muñoz, Markus Krischke, Martin J. Mueller, Nathalie D. Lackus

**Affiliations:** 1 Chair of Pharmaceutical Biology, Julius-Maximilians-University Würzburg, Julius-Von-Sachs-Institute for Biosciences, Julius-Von-Sachs-Platz 2, Würzburg, 97082 Germany; 2 Present address: BOKU University, Department of Biotechnology and Food Science (BTLW), Institute of Bioprocess Science and Engineering (IBSE), Vienna, Austria

**Keywords:** DMNT, Cytochrome P450 monooxygenase, Homoterpene, *Idesia polycarpa*, Salicaceae, Herbivory, *Lymantria dispar*, Volatile organic compounds

## Abstract

**Background:**

Herbivore-induced volatile organic compounds (HIPVs) are important components of plant defense, but the molecular basis of their biosynthesis in woody plants remains poorly understood. Among HIPVs, the homoterpenes (3*E*)-4,8-dimethyl-1,3,7-nonatriene (DMNT) and (*E*,*E*)-4,8,12-trimethyl-1,3,7,11-tridecatetraene (TMTT) are well-characterized herbivore-induced volatiles in herbaceous plants, whereas their biosynthesis in woody species remains largely unexplored. Here, we characterized herbivore-induced volatile emissions from Japanese orange cherry (*Idesia polycarpa*) leaves following feeding by gypsy moth caterpillars (*Lymantria dispar*) and investigated the molecular basis of homoterpene HIPV biosynthesis in this perennial woody species.

**Results:**

Herbivory substantially altered the volatile emission profile of *I. polycarpa* leaves, leading to strong induction of mono- and sesquiterpenes and particularly high emission of the C_11_ homoterpene DMNT, whereas the C_16_ homoterpene TMTT was not detected. These changes were accompanied by elevated jasmonic acid and jasmonate-derived metabolites in damaged leaves. Comparative transcriptome analysis identified a CYP82-family cytochrome P450 monooxygenase and two TPS-g terpene synthases as candidate genes involved in DMNT biosynthesis. Biochemical assays showed that IpCYP82L45 converts (*E*)-nerolidol into DMNT in vitro, while heterologous expression in *Nicotiana benthamiana* demonstrated that IpTPS15 and IpTPS3 produce (*E*)-nerolidol and linalool, respectively. Co-expression of IpTPS15 and IpCYP82L45 in *N. benthamiana* reconstituted DMNT production *in planta*. Furthermore, no diterpene synthase capable of producing (*E*,*E*)-geranyllinalool, the precursor of TMTT, was identified in *I. polycarpa*, consistent with the absence of TMTT emission.

**Conclusions:**

These findings establish a likely jasmonate-associated DMNT biosynthetic pathway in *I. polycarpa* involving the coordinated activity of IpTPS15 and IpCYP82L45. This work advances the molecular understanding of herbivore-induced volatile biosynthesis in non-model woody species and provides new insights into terpene-mediated defense mechanisms in trees.

**Supplementary Information:**

The online version contains supplementary material available at 10.1186/s12870-026-09677-2.

## Introduction

Upon herbivory, plants produce and release a diverse array of volatile organic compounds collectively referred to as herbivore-induced plant volatiles (HIPVs). These compounds contribute to plant defense through multiple mechanisms, including direct deterrence or toxicity toward herbivores, or indirect defense via the recruitment of predators and parasitoids [1–5]. HIPVs are emitted from various plant organs, including leaves and roots, and comprise chemically diverse classes of specialized metabolites, such as aromatic compounds, nitrogen-containing compounds, green leaf volatiles (GLVs), and terpenes [6, 7]. Among these, terpenes represent the most structurally diverse class of plant specialized metabolites and include volatile mono- and sesquiterpenes as well as the irregular homoterpenes (3*E*)−4,8-dimethyl-1,3,7-nonatriene (DMNT) and (*E*,*E*)−4,8,12-trimethyl-1,3,7,11-tridecatetraene (TMTT), widespread occurring volatile compounds [8–10]. Induced DMNT and TMTT emissions play important roles in plant defense and signaling [9, 11]. DMNT and/or TMTT are for instance released as part of herbivore- or elicitor-induced volatile blends in a wide range of herbaceous plant species, including maize (*Zea mays*), sweet potato (*Ipomoea batatas*), and *Arabidopsis thaliana* [12–16]. Beyond their occurrence across diverse plant systems, these homoterpenes have been implicated in multiple layers of plant defense [9]. Direct negative physiological effects on herbivores have been demonstrated for DMNT, including reduced performance of *Plutella xylostella* larvae [17]. In addition, homoterpene emissions contribute to indirect plant defense by mediating tritrophic interactions. For instance, TMTT emission in *Arabidopsis thaliana* enhances indirect defense by attracting the parasitoid wasp *Cotesia rubecula*, which parasitizes *Pieris rapae* larvae and thereby increases plant fitness [14, 16]. Finally, DMNT can also function as a systemic signal. In sweet potato (*I. batatas*), herbivore-induced DMNT emission has been shown to trigger systemic resistance responses, leading to reduced herbivore performance [13]. In addition to aboveground tissues, DMNT formation has also been observed in *A. thaliana* roots upon infection with the oomycete *Pythium irregulare*, where it is implicated in belowground pathogen defense responses [18]. The emission of DMNT and other herbivore-induced volatiles is tightly linked to jasmonate signaling, in which jasmonic acid (JA) and its bioactive conjugate jasmonoyl-isoleucine (JA-Ile) act as central regulators of herbivore-induced metabolic reprogramming in HIPV biosynthesis [19, 20]. Despite its broad ecological relevance, the biosynthetic origin and regulation of homoterpene formation remain incompletely understood, having been characterized in only a limited number of plant species.

Terpenes constitute the largest class of plant specialized metabolites, with more than 60,000 compounds described, and their biosynthesis provides the metabolic framework underlying homoterpene formation [9, 21, 22]. Terpene biosynthesis proceeds via two spatially separated pathways: the cytosolic mevalonate pathway and the plastidial 2-C-methyl-D-erythritol 4-phosphate pathway [23, 24]. Together, these pathways produce the universal C_5_ precursors isopentenyl diphosphate and dimethylallyl diphosphate, which are condensed by isoprenyl diphosphate synthases to generate prenyl diphosphates of defined chain length, including geranyl diphosphate (GPP, C_10_), farnesyl diphosphate (FPP, C_15_), and geranylgeranyl diphosphate (GGPP, C_20_) [25]. These intermediates serve as substrates for terpene synthases (TPSs), which form mono- (C_10_), sesqui- (C_15_), and di- (C_20_) terpenes [8, 26]. Based on their catalytic mechanism, TPSs are classified as class I or class II enzymes [8, 26]. Class I TPSs, including mono- (MTS) and sesquiterpene synthases (STS), catalyze divalent metal ion-dependent ionization of prenyl diphosphates to generate reactive carbocation intermediates that undergo cyclization and rearrangement before termination by deprotonation or nucleophilic attack [8]. These enzymes contain conserved motifs such as the aspartate-rich DDxxD and the NSE/DTE motifs required for metal ion cofactor binding [8]. Class II TPSs, which comprise diterpene synthases (DiTPS), initiate protonation-dependent cyclization of GGPP to form bicyclic prenyl diphosphates such as *ent*-copalyl diphosphate (*ent*-CPP) [26–28]. These intermediates can be further converted by class I DiTPSs into diverse diterpenes, including *ent*-kaurene, and in some cases both activities reside in a single bifunctional enzyme [27, 28]. Further diversification of terpene scaffolds, including homoterpene formation, is mediated by cytochrome P450 monooxygenases [9]. The volatile homoterpenes DMNT (C_11_) and its C_16_ analog TMTT are derived from canonical terpene alcohol precursors via oxidative cleavage reactions [29]. In *Arabidopsis thaliana*, the P450 monooxygenase CYP82G1 converts (*E*)-nerolidol and its C_20_ analog (*E*,*E*)-geranyllinalool into DMNT and TMTT, respectively [29]. Related CYP82 family members have also been implicated in homoterpene biosynthesis in cotton (*Gossypium hirsutum*) and citrus (*Citrus sinensis*) [30, 31].

To date, DMNT homoterpene formation has been characterized primarily in a few herbaceous plant species, including Arabidopsis and cotton, whereas its biosynthesis in long-lived tree species remains largely unexplored, despite the pronounced and persistent biotic pressures faced by perennial plants. The Salicaceae are an important model family for studying defense strategies in woody perennials, with genera such as *Populus* and *Salix* providing key insights into chemical defense [32]. Although salicinoids are recognized as major defense compounds in this family, volatile-mediated responses remain less explored, particularly in species outside the established model systems. The Japanese orange cherry, *Idesia polycarpa*, is a phylogenetically distinct member of the Salicaceae that appears to possess strong constitutive defenses [33], yet little is known about its volatile emission profile. Comparing *I. polycarpa* with the tree model plant black cottonwood (*Populus trichocarpa*) and other Salicaceae thus offers an opportunity to examine interspecific variation in herbivore-induced volatiles and to uncover conserved or lineage-specific mechanisms of HIPV and homoterpene formation in woody perennials.

In the present study, we characterized the herbivore-induced volatile defense response of *I. polycarpa* leaves following feeding by the generalist gypsy moth (*Lymantria dispar*) caterpillars. Herbivory triggered a strong induction of a complex volatile blend dominated by mono- and sesquiterpenes, with the homoterpene DMNT as the most abundant component. Combined transcriptomic and phylogenetic analyses identified a cytochrome P450 monooxygenase from the CYP82 family likely responsible for DMNT formation, as well as a sesquiterpene synthase producing the biosynthetic precursor (*E*)-nerolidol. A corresponding diterpene synthase responsible for (*E*,*E*)-geranyllinalool formation, the precursor in TMTT formation, could not be identified. Heterologous overexpression of the TPS and P450 candidates in *Nicotiana benthamiana* functionally reconstituted DMNT biosynthesis, capturing a key step in the *I. polycarpa* herbivore-induced volatile response.

## Materials and methods

### Plant and insect material

Japanese orange cherry (*Idesia polycarpa*, kindly provided by Christian Paetz, Max Planck Institute for Chemical Ecology) trees were propagated from monoclonal stem cuttings and grown under summer conditions in the greenhouse (day, 23–25 °C; night, 19–23 °C; 50%–60% rel. humidity; 16-h/8-h light/dark cycle) in a 1:1 mixture of sand and clay granules (Klasmann-Deilmann, Geeste, Germany), until they reached about 40 cm in height. Gypsy moth (*Lymantria dispar*) caterpillars were hatched from eggs obtained from Hanna Nadel (US Department of Agriculture, Buzzards Bay, MA, USA) and were reared on an artificial diet (Gypsy moth diet, MP Biomedicals LLC, Illkirch, France).

### Herbivore treatment

Before the start of the experiment, *L. dispar* caterpillars were fed on poplar leaves for one week, and shortly before the start of the experiment, *L. dispar* caterpillars were starved for 13 h. For the herbivore treatment, six *L. dispar* caterpillars (fifth instar) were released on one *I. polycarpa* leaf and allowed to feed for 24 h (8 am to 8 am). On the second day, caterpillars, frass and other caterpillar-derived residues were removed from the plants to avoid volatile contamination, and leaves were enclosed in new PET bags (Bratschlauch, Toppits, Minden, Germany). Volatile collection was then performed from 10 a.m. to 4 p.m. as described in the section “Volatile collection and analysis”. After the volatile collection, leaf material was photographed, harvested, flash-frozen in liquid nitrogen, ground, and one part stored at −80 °C until further processing, while the other part was lyophilized and further stored at room temperature.

### Volatile collection and analysis

Volatiles were collected using a dynamic push–pull system [34]. Air flow was maintained in the system through Teflon tubes. Charcoal filtered air was pumped into the bags at a flow of 0.8 L min^−1^. A portion of the aspirated air (flow: 0.4 L min^–1^) was withdrawn with a second pump and passed through a filter packed with 30 mg Poropak Q (ARS, Inc., Gainesville, FL, USA) to adsorb the volatile compounds. Volatiles were collected for 6 h during the middle of the light period of day 2 after herbivory (see section “Herbivore treatment”). Six biological replicates were measured per treatment. After volatile collection, the volatile compounds were desorbed by eluting the filter with 200 µL dichloromethane containing nonyl acetate as an internal standard (10 ng µL^−1^). Samples were stored at −20 °C until gas chromatography analysis.

Qualitative and quantitative analyses of leaf volatiles were conducted using an Agilent 6890 Series gas chromatograph coupled to an Agilent 5973 quadrupole mass selective detector (Agilent Technologies, Santa Clara, CA, USA; interface temperature, 250 °C; quadrupole temperature, 150 °C; source temperature, 230 °C; electron energy, 70 eV, electron impact ionization) or a flame ionization detector (FID) operated at 300 °C, respectively. The constituents of the volatile bouquet were separated using a ZB5 column (Phenomenex, Aschaffenburg, Germany; 30 m × 0.25 mm × 0.25 µm) and He (MS) or H_2_ (FID) as carrier gas. The sample was injected without split at an initial oven temperature of 45 °C. The temperature was held for 2 min and then increased to 180 °C with a gradient of 6 °C min^−1^, and then further increased to 300 °C with a gradient of 60 °C min^−1^ and a hold of 2 min. Compounds were identified by comparison of retention times and mass spectra to those of authentic standards, by determination of Kovats retention indices or by comparison with reference spectra in the Wiley and National Institute of Standards and Technology (NIST) libraries.

### RNA extraction and reverse transcription

Total RNA was isolated from ground fresh plant material using the InviTrap Spin Plant RNA Mini kit (Invitek, Berlin, Germany) according to manufacturer's instructions. RNA concentration and purity were determined using a spectrophotometer (NanoDrop One, Thermo Fisher, Waltham, MA, USA). RNA was treated with DNaseI (Thermo Fisher Scientific) before complementary DNA (cDNA) synthesis. Single-stranded cDNA was prepared from 1 µg of DNase-treated RNA using SuperScript III reverse transcriptase and oligo (dT_12_-_18_) primers (Invitrogen).

### RNAseq analysis

Total RNA was extracted from leaf material as described above, TruSeqRNA-compatible libraries were prepared, and PolyA enrichment was performed before sequencing. The transcriptomes of three control and three herbivore-treated *I. polycarpa* leaves were sequenced using an Illumina NovaSeq 6000 instrument (Novogene Europe Cambridge, UK) with 20 Mio reads per library, 150 bp, paired end. Trimming of the obtained Illumina reads was performed using the CLC Genomics Workbench (QIAGEN Bioinformatics, Venlo, Netherlands). De novo transcriptome assembly was performed using the CLC Genomics Workbench (QIAGEN) integrated assembly algorithm with the following parameters: bubble size 800, minimum contig length 500 bp, mismatch cost 2, insertion cost 3, deletion cost 3, length fraction 0.8, and similarity fraction 0.9. The completeness of the resulting assemblies was assessed using BUSCO, revealing 63% complete (56% single-copy and 7% duplicated), 19% fragmented, and 18% missing conserved single-copy orthologs. Functional annotation of the assembled transcriptomes was carried out against the SwissProt 2021 database using BLAST (E-value threshold 1.0E − 3, maximum number of hits 20, word size 3, low complexity filter enabled, 40 threads, and HSP length cutoff 33). Trimmed Illumina reads were mapped to the annotated transcriptomes using CLC Genomics Workbench with the following parameters: mismatch cost 2, insertion cost 3, deletion cost 3, length fraction 0.7, similarity fraction 0.9, auto-detection of paired distances enabled, and a maximum of 10 hits per read. Gene expression analysis was performed using the Empirical Analysis of Digital Gene Expression (EDGE) tool implemented in CLC Genomics Workbench, using tagwise dispersion estimates. Differential expression between conditions was assessed using the EDGE test. The EDGE output included raw p-values, fold changes, weighted differences, and multiple-testing corrected p-values (FDR and Bonferroni), which are provided in the Supplementary Data file. For a subset of candidate genes identified based on BLAST annotation, expression levels (RPKM values) were further compared between conditions using Student’s *t*-test as a secondary validation step.

### Identification of putative *I. polycarpa* TPS and CYP82 candidate genes

To identify putative *I. polycarpa* TPS and P450 genes, tBLASTn searches were performed against the de novo assembled transcriptome (see section “*RNA-seq analysis*”) using previously described poplar TPS-g and DiTPS, as well as *A. thaliana* CYP82G1, as query sequences [29, 35–37].

### Phylogenetic analysis and amino acid alignment

A comprehensive alignment of the putative *I. polycarpa* TPS candidate genes together with functionally characterized TPS genes from *Populus trichocarpa* [35–39] was generated using the MUSCLE (codon) algorithm implemented in MEGA11 [40], with the following parameters: gap opening penalty of − 2.9, gap extension penalty of 0, hydrophobicity multiplier of 1.2, and clustering by the UPGMB algorithm. Using the same alignment strategy and parameters, the putative *IpCYP82L45* candidate gene was aligned together with other *CYP82* genes [29, 30]. Tree reconstruction was done with MEGA11 using a Maximum Likelihood algorithm (model/method, Tamura 3-parameter model/General Time Reversible model; substitutions type, amino acid; rates among sites, uniform rates; gaps/missing data treatment, use all sites [41, 42]. A bootstrap resampling analysis with 1000 replicates was performed to evaluate the tree topology. An amino acid alignment of AtCYP82G1 [29], PtCYP82L1 and PtCYP82L2 (https://phytozome-next.jgi.doe.gov/) with IpCYP82L45 was performed with MEGA11 [40] and visualized with BioEdit [43].

### Cloning of identified candidate genes

The open reading frame of the IpTPS candidate gene, *IpTPS19*, was amplified from herbivore-induced *I. polycarpa* leaf cDNA and cloned into the pOPINF vector for heterologous expression in *Escherichia coli*, using the full-length sequence as determined by in silico subcellular localization predictions (Supplemental Table S2). The previously characterized *P. trichocarpa* TPS genes *PtTPS17* and *PtTPS19* [37] were amplified from poplar cDNA as truncated forms (Δ195nt and Δ126nt, respectively) and cloned into pOPINF. The P450 candidate *IpCYP82L45* was amplified from herbivore-induced *I. polycarpa* cDNA, and *AtCYP82G1* from *A. thaliana* cDNA; and both were cloned as full-length sequences into the pESC-Leu2d vector for heterologous expression in *Saccharomyces cerevisiae*. For transient overexpression in *Nicotiana benthamiana*, full-length coding sequences of *IpTPS3*, *IpTPS15*, *IpTPS19*, *IpCYP82L45*, *AtCYP82G1*, and *PtTPS10* [36] were cloned into the pCAMBiA 2300U vector. Oligonucleotide sequences are provided in Supplementary Table S4. All constructs were verified by full-length sequencing.

### Heterologous expression in *Escherichia coli* and IpTPS enzyme assays

The *E. coli* strain BL21 Star™ (DE3) (New England Biolabs, Ipswich, MA, USA) was used for expression of *IpTPS* candidates cloned in the expression vector pOPINF. Cultures were grown at 37 °C, induced at an OD_600_ = 0.6 with 1 mM IPTG, subsequently placed at 18 °C and grown for another 16 h. Cells were collected by centrifugation (10 min, 5,000 g, 4 °C) and disrupted by sonication (4 × 30 s; Labsonic®, Sartorius Stedim Biotech SA, France) in chilled extraction buffer (10 mM Tris–HCl (pH 7.5), 1 mM dithiothreitol, 10% (v/v) glycerol). Cell fragments were removed by centrifugation (30 min, 16,000 rpm, 4 °C) and supernatant was desalted by passage through Illustra NAP-5 columns (Cytiva, Marlborough, MA, USA).

Enzymatic activity was determined by enzyme assays containing 50 µl of the bacterial raw extract, 44 µl extraction buffer, 10 mM MgCl_2_ and 1 µg substrate (GPP, (*E,E*)-FPP or *(E,E,E*)-GGPP, Merck KGaA, Darmstadt, Germany) in a 1.5 ml Teflon-sealed, screw capped glass vial. To determine enzymatic activity for the substrates GPP and (*E,E*)-FPP a SPME (solid phase microextraction) fiber consisting of 100 μm polydimethylsiloxane (SUPELCO, Belafonte, PA, USA) was placed into the headspace of the vial for one hour incubation at 30 °C. For analysis of the adsorbed reaction products, the SPME fiber was directly inserted into the injector of a GC–MS system (see “Analysis of in vitro and in planta enzyme products”). Assays with *(E,E,E*)-GGPP were overlayed with 100 µl *n*-hexane and incubated for one hour at 30 °C, *n*-hexane phase was taken and analyzed using a GC–MS system (see “Analysis of in vitro and in planta enzyme products”). Coupled enzyme assays combining *PtTPS17* with either *PtTPS19* or *IpTPS19* were performed as described above. Equal volumes of bacterial crude extracts were mixed and incubated under the same reaction conditions and buffer composition used for the individual enzyme assays with the substrate *(E,E,E*)-GGPP. Crude extracts from *Escherichia coli* carrying the empty vector served as negative controls and were assayed with the respective substrates under identical conditions.

### Heterologous expression of IpCYP82L45 in *Saccharomyces cerevisiae*

For heterologous overexpression of the identified P450 candidate and the already described AtCYP82G1 [29], constructs generated in the pESC-Leu2d vector (as described above) were transformed into *Saccharomyces cerevisiae* strain WAT11 using the Frozen-EZ Yeast Transformation II Kit (Zymo Research) following the manufacturer’s instructions. Single transformants were selected and used to inoculate 20 mL starter cultures of synthetic complete medium lacking L-Leu (SC-Leu; yeast nitrogen base without amino acids/with ammonium sulfate, yeast synthetic drop-out medium without leucine (Merck KGaA), adenine hemisulfate) containing 2% glucose. Cultures were grown overnight at 28 °C with shaking at 180 rpm. Cells corresponding to an OD_600_ of 1.0 were transferred into 100 mL YPDA medium supplemented with 2% glucose and incubated for an additional 30–35 h at 28 °C and 180 rpm. Cultures were then harvested by centrifugation (5,000 × g, 16 °C, 5 min), and pellets were resuspended in 100 mL YPDA medium containing 2% galactose to induce recombinant protein expression. Induction was carried out for 15–18 h at 25 °C and 160 rpm.

### Microsome preparation

Microsomes were isolated following the protocol described by [44]. Briefly, induced cultures were centrifuged (7,500 × g, 10 min, 4 °C), and the resulting pellet was resuspended in 30 mL TEK buffer (50 mM Tris–HCl, 1 mM EDTA, 100 mM KCl, pH 7.5) and centrifuged again under the same conditions. The pellet was then resuspended in 2 mL TES buffer (50 mM Tris–HCl, 1 mM EDTA, 600 mM sorbitol, 10 g/L BSA, and 1.5 mM β-mercaptoethanol per 100 mL, pH 7.5) and transferred to a 50-mL conical tube. Glass beads (0.45–0.50 mm diameter) were added to match the suspension volume. Cells were lysed by five cycles of vigorous manual shaking for 1 min, each followed by cooling on ice for 1 min. The crude extract was collected by rinsing the glass beads four times with 5 mL TES buffer. Combined wash fractions were centrifuged (7,500 × g, 10 min, 4 °C), and the resulting supernatant was subjected to ultracentrifugation (100,000 × g, 90 min, 4 °C). The microsomal pellet was gently homogenized in 2 mL TEG buffer (50 mM Tris–HCl, 1 mM EDTA, 30% glycerol) using a Potter–Elvehjem glass homogenizer (Fisher Scientific). Microsomal preparations were aliquoted and stored at −20 °C until use in subsequent in vitro assays.

### Microsome in vitro analysis of recombinant IpCYP82L45 activity

To evaluate the enzymatic activities of the P450 candidate IpCYP82L45, microsomal preparations containing the recombinant protein were incubated with a set of potential substrates. Enzyme assays were generally performed in glass vials containing 200 µL of reaction mixture composed of 50 mM sodium phosphate buffer (pH 7.0), 100 µM substrate, 1 mM NADPH, and 78 µL of microsomes. Reactions were incubated for 2 h at 30 °C and 300 rpm shaking. To determine enzymatic activity, a solid-phase microextraction (SPME) fiber (100 µm polydimethylsiloxane) was subsequently inserted into the headspace of the respective assays and incubated at room temperature for an additional hour to adsorb volatile enzymatic products. The SPME fibers were introduced into the injector of the GC–MS system, and product formation was analyzed as described in “Analysis of in vitro and in planta enzyme products”. The substrates tested included (*E*)-nerolidol, (*Z*)-nerolidol, and (*E*,*E*)-geranyllinalool. Negative controls included assays conducted without substrate, without microsomes, with denatured microsomes, and without cofactor. Microsomes expressing AtCYP82G1 served as positive controls.

### Transient overexpression of *IpTPS* and* IpCYP82L45* genes in *Nicotiana benthamiana*

For gene expression in *N. benthamiana*, obtained *IpTPS, IpCYP82L45*, *AtCYP82G1*, and *PtTPS10* pCAMBiA 2300U constructs (see “Cloning of identified candidate genes”), *eGFP* and *p19::pBIN* constructs were separately transformed into *Agrobacterium tumefaciens* strain GV3101. Liquid cultures of transformed *A. tumefaciens* were grown at 28 °C for two days at 200 rpm, after incubation pelleted by centrifugation (4000 g, 14 °C, 20 min), respective pellets resuspended in infection medium (10 mM MES (pH 5.7), 10 mM MgCl_2_, 100 µM acetosyringone) to an OD_600_ of 0.4 to 0.6, and further incubated at room temperature for additional 1–3 h at 200 rpm. For infiltration, equal volumes of the constructs-of-interest and the *p19::pBIN* vector *A. tumefaciens* cultures were mixed and infiltrated with a 1 ml syringe into the lower side of all leaves of 3–4 week-old *N. benthamiana* plants. Transiently transformed plants were kept for one night in the dark and were then transferred to the greenhouse. Volatile sampling and analysis of the *N. benthamiana* plants is described in section “Analysis of in vitro and in planta enzyme products”.

### n-hexane extraction of plant material

To determine diterpene accumulation in *N. benthamiana* leaves, 100 mg of fresh, powdered plant material was extracted in a GC glass vial with 300 μL of hexane containing 10 ng/μL nonyl acetate as an internal standard. Extracts were shaken at 450 rpm overnight at room temperature. Following centrifugation for 10 min at 5,000xg, the supernatant was collected and analyzed by GC–MS and GC-FID as described in the section ‘*Analysis of *in vitro* and in planta enzyme products*’.

### Analysis of in vitro and in planta enzyme products

In vitro TPS enzyme activity was analyzed using an Agilent 6890 N series gas chromatograph coupled to an Agilent 5975 quadrupole mass selective detector (interface temperature, 250 °C; quadrupole temperature, 150 °C; source temperature, 230 °C; electron energy, 70 eV, electron impact ionization). Compounds were separated using a DB5 column (OPTIMA 5 MS, Macherey–Nagel, Düren, Germany; 30 m × 0.25 mm × 0.25 µm) and He as carrier gas. For monoterpene analysis, the chromatographic separation was achieved by the following gradient: the temperature was held at 45 °C for 2 min, increased with a gradient of 7 °C min^−1^ to 130 °C, and then further increased to 300 °C with a gradient of 100 °C min^−1^ and a hold of 1 min. For the analysis of sesquiterpene formation, chromatographic separation was achieved using a gradient starting at 80 °C with a hold time of 2 min, followed by an increase to 180 °C at a rate of 7 °C min^−1^, continued by an increase of 100 °C min^−1^ to 300 °C, and a final hold time of 1 min. To determine diterpene formation, the temperature gradient started at an initial oven temperature of 80 °C for 2 min, followed by an increase to 280 °C at a rate of 5 °C min^−1^, and then further increased to 320 °C at a rate of 100 °C min^−1^ and a hold time of 2 min. For analysis of IpCYP82L45 enzymatic activity (see section “Microsome in vitro analysis of recombinant IpCYP82L45 activity”), analysis was conducted using the GC–MS system, setup and column as described above and chromatographic parameters used for separation as described in the section "Volatile collection and analysis". Enzymatic products were identified by comparison of retention times and mass spectra with those of authentic standards, by comparison of respective positive controls from previously characterized enzymes or by comparison with reference spectra in the Wiley and NIST libraries.

Volatiles from transiently transformed *N. benthamiana* plants were sampled using a dynamic push–pull system with the settings described above in the section "Volatile collection and analysis", with the following exceptions: Volatiles were collected for 24 h from the fourth to the fifth day after transformation from whole *N. benthamiana* plants packed in PET bags (Bratschlauch, Toppits, Minden, Germany). The soil pots of the *N. benthamiana* plants were previously wrapped in aluminum foil to avoid volatile contamination. After sampling, volatiles were desorbed by eluting the filter with 200 µL of dichloromethane containing nonyl acetate as internal standard (10 ng µL^−1^). Samples were stored at −20 °C until gas chromatographic analysis. The respective transformed leaf material was harvested and weighted for quantification of emitted volatiles. For each construct combination, three biological replicates were investigated. The volatile sample was injected without splitting using the GC–MS system, setup and column as described above for the in vitro analysis and chromatographic parameters used for separation as described in the section "Volatile collection and analysis". Volatiles were identified by comparison of retention times and mass spectra with those of authentic standards, by comparison of respective positive controls from previously characterized enzymes or by comparison with reference spectra in the Wiley and NIST libraries. In addition, to quantify the emitted volatiles from the *N. benthamiana* plants, the samples were measured and analyzed using a GC-FID (Agilent 6850 Series II System, Agilent Technologies, Santa Clara, CA, USA) system operating at 250 °C with H_2_ as carrier gas. Chromatographic separation was performed using a DB5 column (OPTIMA 5 MS, Macherey–Nagel, Düren, Germany; 30 m × 0.25 mm × 0.25 µm) and oven parameters as described in "Volatile collection and analysis".

### Quantification of jasmonic acid, JA-Ile conjugates, OPDA, ABA and salicylic acid

For the quantification of jasmonic acid (JA), jasmonic acid-isoleucine conjugates (JA-Ile conjugates), 12-oxophytodienoic acid (OPDA), abscisic acid (ABA) and salicylic acid (SA), 10 mg of lyophilized *I. polycarpa* leaf material was used and extracted with 950 µl of ethyl acetate: formic acid (99:1; v/v) and additional 50 µl of internal standard (dihydro-JA, [^18^O_2_]-OPDA, JA-norvaline conjugate, [D_4_]-SA and [D_6_]-ABA; 0.1 ng/µl each in acetonitrile (SUPELCO, Bellefonte, PA, USA)). Samples were homogenized in a ball mill (3 min, 20 Hz, Retsch MM 400 mixer mill), centrifuged (20 min, 14,000 rpm), and the supernatant taken. The supernatant was dried (45 °C; 1500 min^−1^) and the remaining pellet dissolved in 40 µl 1:1 acetonitrile:water (v/v). Upon further centrifugation (10 min, 14,000 rpm) the supernatant was taken and analyzed by UPLC-ESI–MS/MS using a Waters Acquity I-Class ultra-high-performance liquid chromatography system (Milford, MA, USA) coupled to an AB Sciex 6500 + QTRAP® tandem mass spectrometer (AB Sciex, Framingham, MA, USA). Separation was achieved on a Acquity UPLC BEH RP C18 column (2.1 × 50 mm, 1.7 µm particle size, Waters, Milford, MA, USA). Formic acid (0.1%) in water (v/v) and acetonitrile were employed as mobile phases A and B, respectively. The elution profile was: 0–7 min, 3–100% B; 7–9 min 100% B; 9–12 min 3% B. The mobile phase flow rate was 0.25 ml min^−1^, and the column temperature maintained at 40 °C. The ion spray voltage was maintained at 4.0 kV in a negative ionization mode, curtain gas was kept at 30 psi and nebulizer gas (GS1) and drying gas (GS2) were adjusted to 50 and 80 psi, respectively, at a temperature of 600 °C. Selected reaction monitoring (SRM) was used to monitor analyte parent ion → product ion formation as follows: *m/z* 137 → 93 (declustering potential (DP) −25 V, collision energy (CE) −22 V) for salicylic acid; *m/z* 141 → 97 (DP −10 V, CE −22 V) for D_4_-salicylic acid; *m/z* 263 → 153 (DP −40 V, CE −14 V) for abscisic acid; *m/z* 269 → 159 (DP −25 V, CE −14 V) for D_6_-abscisic acid; *m/z* 209 → 59 (DP −35 V, CE −14 V) for jasmonic acid; *m/z* 322 → 130 (DP −55 V, CE −38 V) for jasmonic acid-isoleucine conjugates; *m/z* 211 → 59 (DP −35 V, CE −16 V) for dihydro-jasmonic acid; *m/z* 308 → 116 (DP −60 V, CE −26 V) for jasmonic acid-norvaline conjugate; *m/z* 291 → 165 (DP −85 V, CE −26 V) for 12-oxophytodienoic acid; *m*/*z* 295 → 165 (DP −55 V, CE −26 V) for [1,1-^18^O_2_] 12-oxo-phytodienoic acid.

### Statistical analysis

Throughout the manuscript, data are presented as means ± SE, or as median with interquartile range and outliers. To assess statistical significance Student’s t-tests or Mann–Whitney Rank Sum Tests were performed with SigmaPlot 14.0 for Windows (Systat Software Inc., https://systatsoftware.com) or with RStudio (version 2023.12.1–402, R Development Core Team, http://R-project.org). Whenever necessary, the data were log transformed to meet statistical assumptions such as normality and homogeneity of variances. The statistical analyses performed are displayed for each data set in the respective figure and table legends.

### Availability of data and materials and accession numbers

All supporting data are included as additional files. Constructs described in this work and datasets analyzed during the current study are available from the corresponding author upon request. Sequence data for *IpTPS3* (PZ213723), *IpTPS15* (PZ213724), *IpTPS19* (PZ213722), and *IpCYP82L45* (PZ213725) can be found in the NCBI GenBank (https://www.ncbi.nlm.nih.gov/genbank/) under the corresponding identifiers. The RNA sequencing data generated in this study have been deposited in the NCBI Sequence Read Archive (SRA) under BioProject accession number PRJNA1473663. Associated BioSample accession numbers are SAMN60567041–SAMN60567046. Individual SRA run accession numbers are SRR38967400–SRR38967405.

### Declaration of generative AI and AI-assisted technologies in the writing process

During the preparation of this work, the authors used ChatGPT and DeepL in order to improve the clarity and accuracy of the English language. After using this tool/service, the authors reviewed and edited the content as needed and take full responsibility for the content of the publication.

## Results

### The herbivore-induced volatile blend of *I. polycarpa* leaves is characterized by DMNT and other volatile mono- and sesquiterpenes

**Fig. 1:**
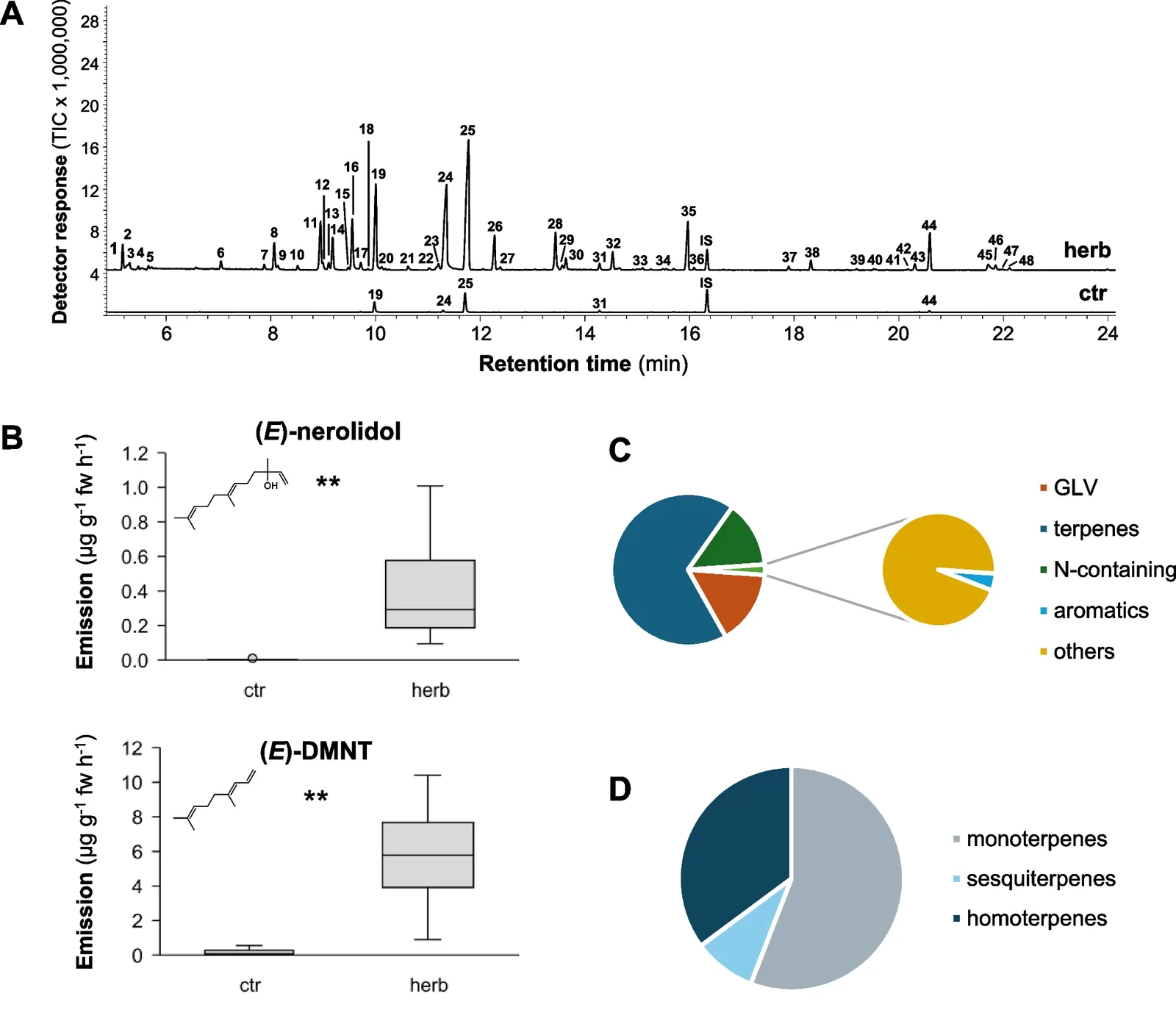
Volatiles emitted by undamaged (ctr) and gypsy moth caterpillar (*Lymantria dispar,* herb*)* damaged Japanese orange cherry (*Idesia polycarpa*) leaves. **A** Representative volatile emission profiles of undamaged control leaves and leaves subjected to herbivore feeding. **B** Quantitative analysis of selected terpene compounds emitted from control and herbivore-damaged leaves. **C** Relative contributions of major volatile compound classes to the total volatile blend. **D** Relative contributions of mono-, sesqui- and homoterpenes to the total terpenoid emission. Volatile compounds were collected using a push–pull system and subsequently analyzed and quantified by gas chromatography–mass spectrometry (GC–MS) and gas chromatography–flame ionization detection (GC–FID), respectively. Box plots show median, interquartile range, and outliers (*n* = 6 biological replicates). 1, (*E*)−2-hexanal; 2, 3-methylbutyraldoxime isomer 1; 3, 2-methylbutyraldoxime isomer 1; 4, 2-methylbutyraldoxime isomer 2 and (*E*)−2-hexanal; 5, 3-methylbutyraldoxime isomer; 6, α-pinene*; 7, methyl 2-hexenoate; 8, sabinene; 9, β-pinene*; 10, myrcene*; 11, (*Z*)−3-hexenyl acetate; 12, unidentified green leaf volatile 1; 13, hexyl acetate; 14, (*E*)−2-hexenyl acetate; 15; β-phellandrene; 16, 1,8-cineole; 17, (*Z*)-β-ocimene; 18, phenylacetaldehyde; 19, (*E*)-β-ocimene*; 20, unidentified monoterpene 1; 21, (*Z*)-linalool oxide; 22, (*E*)-linalool oxide; 23, (*Z*)-DMNT; 24, linalool*; 25, (*E*)-DMNT^#^; 26, benzyl cyanide; 27, (*Z*)−3-hexenyl butyrate and *cis*-epoxy-β-ocimene; 28, *cis*−3-hexenyl isobutyrate; 29, unidentified monoterpene 2; 30, unidentified compound 1; 31, unidentified compound 2; 32, *cis*−3-hexenyl-2-methylbutanoate; 33, phenylacetaldoxime isomer 1; 34, phenylacetaldoxime isomer 2; 35, indole; 36, 2-phenylnitroethane; IS, internal standard; 37, (*Z*)−3-hexenyl hexanoate; 38, *cis*-jasmone; 39, unidentified compound 3; 40, (*E*)-β-farnesene*; 41, germacrene D; 42, unidentified compound 4; 43; (*Z,E*)-α-farnesene, 44; (*E,E*)-α-farnesene, 45; (*E*)-nerolidol*, 46, (*Z*)−3-hexenyl benzoate; 47, unidentified compound 5; 48, (*E*)−3-hexenyl benzoate. Compounds marked with ‘*’ were identified by comparison of retention time and mass spectrum to those of authentic standards. Compound marked with ‘^#^’ by comparison of retention time and mass spectrum to those of the previously described P450 enzyme AtCYP82G1 [23]. Other compounds were identified by database comparisons. Differences between treatments were analyzed by a Student’s *t*-test or Mann–Whitney-U test (* *P* < 0.05; ** *P* < 0.01; *** *P* < 0.001). (*E*)-DMNT (*P* = 0.002; T = 21.000); (*E*)-nerolidol (P = 0.002; T = 21.000). (*E*)-DMNT, (3*E*)−4,8-dimethyl-1,3,7-nonatriene; fw, fresh weight; nd, not detected

To characterize herbivore-induced defense responses in *I. polycarpa* leaves, volatile organic compound (VOC) emissions were analyzed following feeding by caterpillars of the generalist gypsy moth (*Lymantria dispar*) and compared with undamaged controls. Volatiles were collected using an active push–pull system and analyzed by GC–MS and GC-FID. Herbivory caused pronounced quantitative and qualitative changes in the VOC profile, with significantly induced emission of 45 compounds relative to control leaves (Fig. 1A; Supplemental Table S1). The induced blend was dominated by terpenoids, nitrogen-containing compounds, and green leaf volatiles (GLVs), whereas aromatics were present only in minor amounts (Fig. 1A; Supplemental Table S1). Nitrogen-containing compounds included indole, 2-phenylnitroethane, and benzyl cyanide, while GLVs compounds included multiple components, such as *trans*−2-hexenyl acetate (Fig. 1A; Supplemental Table S1). Terpenoids were the most abundant class, accounting for 68% of total emissions and 20 of the 48 compounds identified (Fig. 1C; Supplemental Table S1). The terpenoid fraction included monoterpenes such as (*E*)-β-ocimene, 1,8-cineole, and linalool, and sesquiterpenes, including (*E*,*E*)-α-farnesene and (*E*)-nerolidol (Fig. 1A/B; Supplemental Table S1). Notably, the homoterpene DMNT was strongly induced, representing the most abundant compound and contributing to 35% of the total terpenoid emissions (Fig. 1B-D; Supplemental Table S1). However, its C_16_ analog TMTT was not detected in the herbivore-induced volatile blend. These results indicate that herbivory by *L. dispar* triggers a terpene-dominated volatile response in *I. polycarpa* leaves, with DMNT being the most strongly induced and abundant volatile.

### Herbivore-induced jasmonate accumulation in* I. polycarpa* leaves

**Fig. 2:**
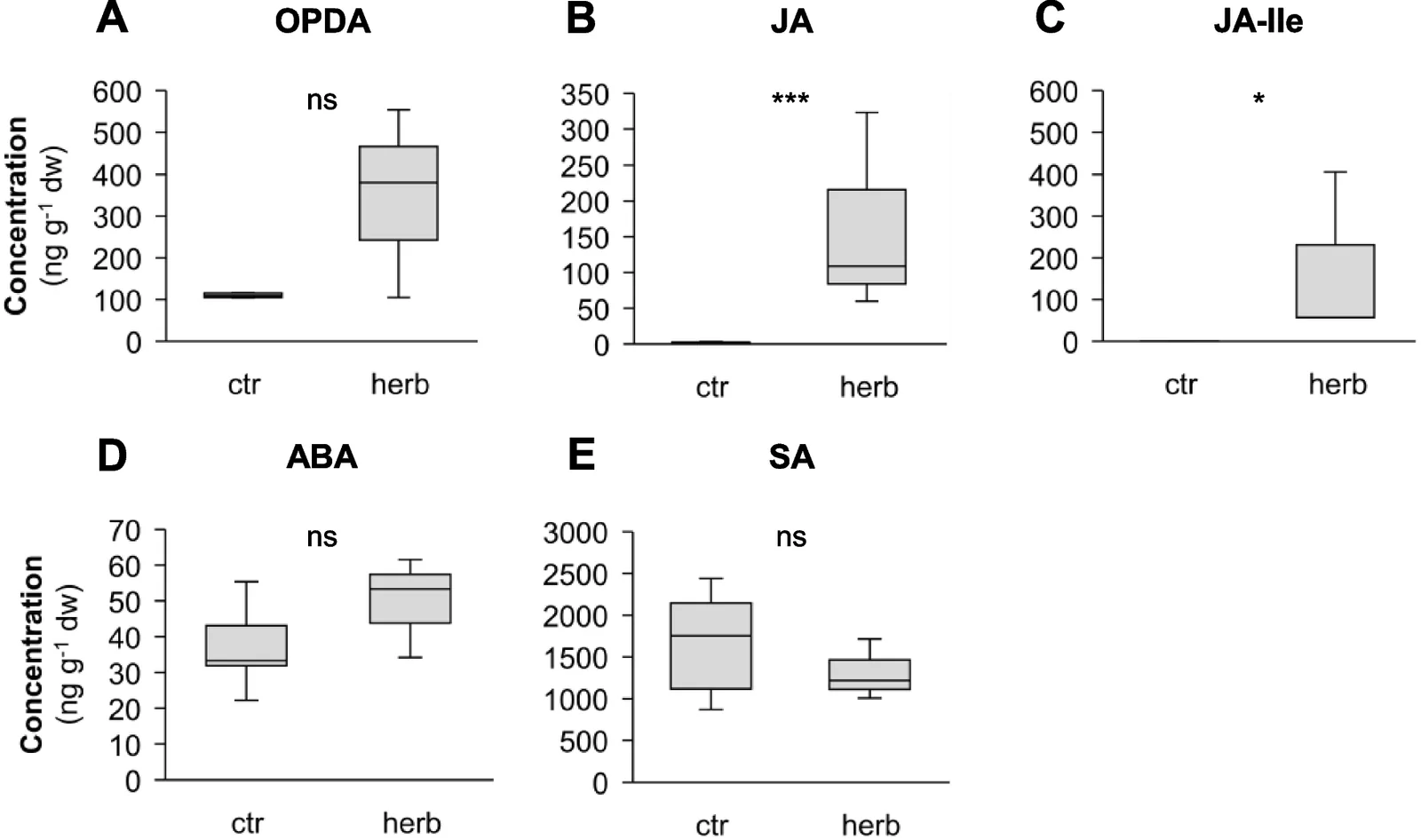
Phytohormone content in undamaged (ctr) and gypsy moth caterpillar (*Lymantria dispar,* herb*)* damaged Japanese orange cherry (*Idesia polycarpa*) leaves. Compounds were extracted with ethyl acetate: formic acid (*v*:*v*; 99:1) from lyophilized plant material and analyzed using LC–MS/MS. Box plots show median, interquartile range, and outliers (n = 3–6). Differences between treatments were analyzed by a Student’s *t*-test or Mann–Whitney-U test (* *P* < 0.05; ** *P* < 0.01; *** *P* < 0.001). OPDA (*P* = 0.1033; t = −1.8726); JA (*P* = 0.0002; t = −7.0762); JA-Ile (*P* = 0.02819; W = 0.00); ABA (P = 0.1948; t = −1.4335); SA (P = 0.4189; t = 0.85868). ABA, abscisic acid; dw, dry weight; JA, jasmonic acid; JA-Ile, jasmonic acid isoleucine conjugates; ns, not significant; OPDA, 12-oxo-phytodienoic acid; SA, salicylic acid

To assess the impact of herbivory on phytohormone dynamics in *I. polycarpa* leaves, we quantified jasmonic acid (JA), jasmonic acid–isoleucine (JA-Ile), the JA biosynthetic precursor 12-oxo-phytodienoic acid (OPDA), abscisic acid (ABA), and salicylic acid (SA). Herbivory resulted in a strong and significant accumulation of JA and JA-Ile, which increased approximately 70-fold and 300-fold, respectively, compared with control leaves (Fig. 2 B/C). In contrast, OPDA levels were not significantly affected by herbivory, although OPDA was present at the highest concentrations among the JA-related metabolites in both control and herbivore-treated leaves (Fig. 2 A). Moreover, no significant changes in ABA or SA levels were observed in response to herbivory (Fig. 2 D/E), whereas SA accumulated at the highest absolute concentrations among all phytohormones analyzed (Fig. 2 E). Overall, herbivory specifically induced JA and JA-Ile, jasmonates that are typically associated with biotic stress responses, while OPDA, ABA, and SA levels remained unchanged.

### Integrated transcriptomic and in silico analyses identify herbivory-responsive CYP82 and terpene synthase candidates in *I. polycarpa*

**Fig. 3:**
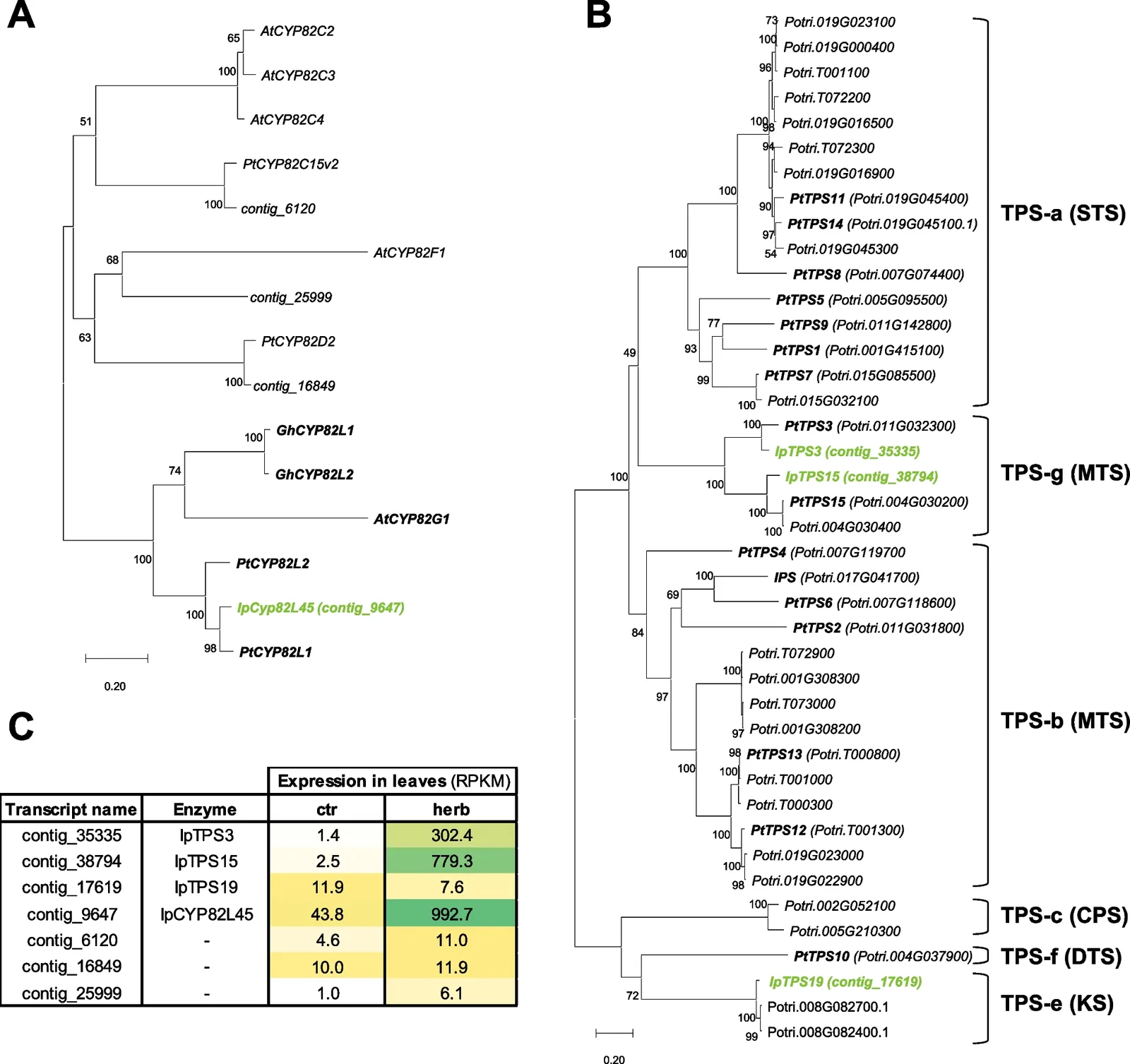
Identification of Japanese orange cherry (*Idesia polycarpa)* cytochrome P450 monooxygenases belonging to the CYP82 family and tentative terpene synthases. **A** Phylogenetic analysis of *I. polycarpa* (*Ip*) *CYP82L45* and *CYP82* members from other plants. **B** Phylogenetic analysis of putative *I. polycarpa terpene synthases* (*TPS*) and *TPS* from black cottonwood (*Populus trichocarpa*, Pt). The trees were inferred by using the Maximum Likelihood method based on the Tamura 3-parameter model [47] (A) or the General Time Reversible model [48] **B** implemented in MEGA11 [46]. Bootstrap values (n = 1000) are shown next to each node. The trees are drawn to scale, with branch lengths measured in the number of substitutions per site. Codon positions included were 1 st + 2nd + 3rd + Noncoding. *IpCYP82L45* and *IpTPS* candidate genes further considered in this study are highlighted in green. *PtTPS and CYP82* in bold have previously been described in [23, 24, 28–30, 44, 45]. TPS-a to h represent TPS subfamilies [33]. **C** Expression of *CYP82* and *TPS* candidate genes in gypsy moth (*Lymantria dispar*) caterpillar-damaged (herb) and undamaged (ctr) leaves of *I. polycarpa*. Leaf transcriptomes were sequenced and reads mapped to the de novo assembled transcriptome. Mean expression (*n* = 3 biological replicates) is shown. Fold changes and *P*-values are given in Supplemental Table S3. At, *Arabidopsis thaliana*; CDS, copalyl diphosphate synthase; DTS, diterpene synthase; Gh, *Gossypium hirsutum*; IPS, isoprene synthase; KS, kaurene synthase; MTS, monoterpene synthase; Pt, *Populus trichocarpa*; RPKM, reads per kilobase per million mapped reads; STS, sesquiterpene synthase

To investigate the molecular basis of herbivore-induced volatile formation in *I. polycarpa* leaves, we performed comparative RNA-seq analyses on control and herbivore-treated tissue and generated a de novo transcriptome assembly. The assembly was screened for TPS-g and DiTPS candidates responsible for producing the sesquiterpene and diterpene alcohols (*E*)-nerolidol and (*E*,*E*)-geranyllinalool, the biosynthetic precursors of DMNT and TMTT, respectively. Using tBLASTn searches with functionally characterized TPS-g proteins from *Populus trichocarpa* as queries [35, 36], we identified two full-length TPS-g candidate sequences in the *I. polycarpa* transcriptome (contig_35335 and contig_38794). A single DiTPS candidate (*contig_17619*) was identified using tBLASTn with described *P. trichocarpa* DiTPS from the TPS-e/-f and TPS-c clades as query sequences [36, 37]. Phylogenetic analysis together with the poplar TPS family members confirmed that *contig_35335* and *contig_38794* clustered within the TPS-g family (Fig. 3B), and were thus designated as *IpTPS3* and *IpTPS15*, respectively, following the numbering of their closest poplar orthologues (Fig. 3B) [35, 36]. The DiTPS candidate *contig_17619* clustered within the TPS-e clade of kaurene synthases and was designated as *IpTPS19* according to its poplar orthologue (Fig. 3B). All three candidates possess canonical class I TPS motifs, including the aspartate-rich DDxxD motif and the NSE/DTE motif (Supplemental Table S2). For IpTPS3 and IpTPS15, the second arginine of the RxR motif is replaced by glutamine, and the RR(x)_8_W motif is absent, as it is also the case for IpTPS19 (Supplemental Table S2). Notably, IpTPS19 contains a conserved SxYDTxW motif characteristic of kaurene synthases and copalyl diphosphate synthases (Supplemental Table S2, [45]). Subcellular localization predictions using TargetP 2.0 (https://services.healthtech.dtu.dk/services/TargetP-2.0/) [32]) indicated a plastidial targeting for IpTPS3, but not for IpTPS15 or IpTPS19 (Supplemental Table S2). Gene expression analysis revealed strong herbivore-induced upregulation of the TPS-g candidates, with 209.8- and 309.5-fold increases for *IpTPS3* and *IpTPS15*, respectively, whereas expression of the DiTPS *IpTPS19* remained almost unaffected by herbivory (Fig. 3C, Supplemental Table S3, Supplemental Data File 2).

To identify genes underlying DMNT biosynthesis in *I. polycarpa*, the de novo assembly was screened for CYP82 homologs using tBLASTn with *Arabidopsis thaliana* CYP82G1 (AtCYP82G1) as query sequence, a previously described DMNT and TMTT synthase [29]. This analysis identified four putative CYP82 homologs. Phylogenetic reconstruction indicated that one candidate, *contig_9647*, clustered closely with *AtCYP82G1* and the characterized cotton *CYP82* involved in homoterpene formation, whereas the remaining three candidates (*contig_6120*, *contig_16849*, *contig_25999*) grouped with other *Populus trichocarpa CYP82* family members (Fig. 3A). Transcript abundance analysis showed that gene expression of *contig_9647* was strongly upregulated by herbivory (22.6-fold) (Fig. 3C, Supplemental Table S3, Supplemental Data File 2), whereas the other candidates (*contig_6120*, *contig_16849*, *contig_25999*) had low basal expression and were only weakly induced (Fig. 3C, Supplemental Table S3, Supplemental Data File 2). The gene expression profile of *contig_9647* closely mirrored herbivore-induced DMNT emission and, together with the phylogenetic analysis, supports its assignment as the candidate most likely responsible for DMNT biosynthesis (Figs. 1; and 3C). Accordingly, this candidate was further analyzed in the study and designated as *IpCYP82L45* (kindly assigned by D. Nelson, University of Tennessee). In silico analysis and an amino acid alignment confirmed that IpCYP82L45 possesses conserved cytochrome P450 features, including the proline-rich region, PERF motif, heme-binding domain, and six predicted substrate recognition sites previously implicated in homoterpene formation (Supplemental Fig. S1, [29]).

### Functional characterization identifies IpTPS3 and IpTPS15 as linalool and (E)-nerolidol synthases, respectively, and IpTPS19 as an *ent*-kaurene synthase

**Fig. 4:**
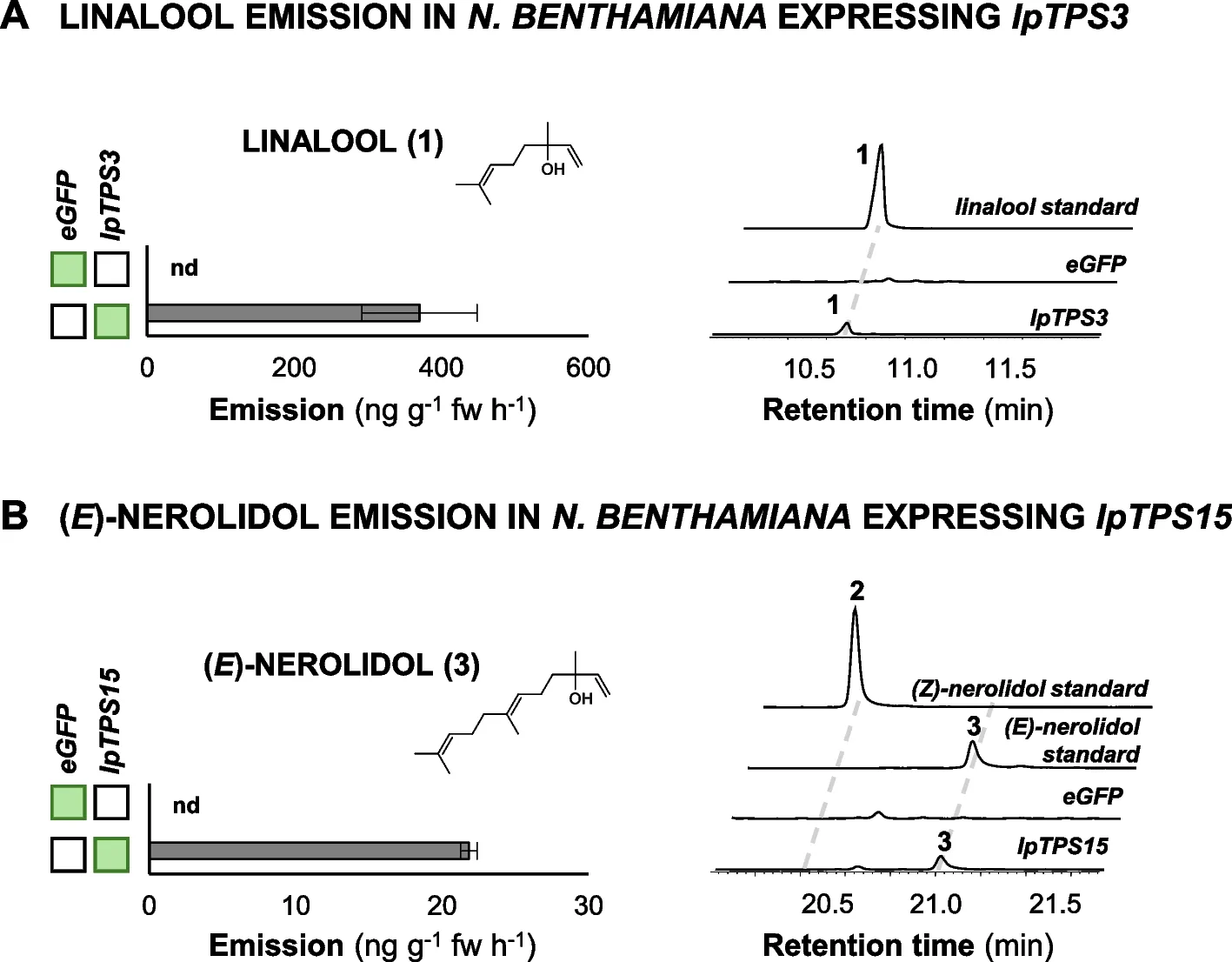
Volatile emissions from *Nicotiana benthamiana* plants transiently overexpressing *Idesia polycarpa* candidate terpene synthases (IpTPS). Volatile emissions and representative GC-chromatograms are shown for IpTPS3 (**A**), and IpTPS15 (**B**). Plants were infiltrated with *Agrobacterium tumefaciens* carrying *35S:eGFP*, *35S:IpTPS3*, or *35S:IpTPS15*. Volatiles were collected four days after infiltration over 24 h using an active push–pull system and analyzed by gas chromatography–mass spectrometry (GC–MS) and quantified using gas chromatography–flame ionization detection (GC–FID). Data are presented as means ± SE (n = 3 biological replicates). fw, fresh weight; nd, not detected. 1, linalool*; 2, (*Z*)-nerolidol*; 3, (*E*)-nerolidol*. Compounds marked with ‘*’ were identified by comparison of retention time and mass spectrum to authentic standards. Other compounds were identified via database comparisons

For enzymatic characterization, full-length IpTPS constructs were transiently expressed in *Nicotiana benthamiana* leaves using *Agrobacterium tumefaciens* to assess their activity *in planta*. Co-infiltration with the p19 silencing suppressor enhanced expression, while an eGFP construct served as a negative control. Volatile compounds released from the transformed leaves were collected and analyzed by GC–MS and GC–FID to profile the terpenes generated. Transient expression of individual *IpTPS* candidates revealed that *IpTPS3* and *IpTPS15* encode functional terpene synthases (Fig. 4). Leaves overexpressing *IpTPS3* emitted high levels of the monoterpene alcohol linalool (Fig. 4A), whereas expression of *IpTPS15* resulted in strong emission of the sesquiterpene alcohol (*E*)-nerolidol (Fig. 4B). Transient expression of *IpTPS19*, however, did not alter the volatile profile relative to controls (data not shown). Considering the lower volatility of diterpenes compared with mono- and sesquiterpenes, leaf tissue was additionally extracted with *n*-hexane to assess potential accumulation of less volatile products; nevertheless, neither geranyllinalool nor other putative diterpenes were detected in *IpTPS19*-expressing *N. benthamiana* leaves (Supplemental Fig. S2). By comparison, leaves expressing the previously characterized *Populus trichocarpa* geranyllinalool synthase PtTPS10 [36] accumulated the expected product, confirming the suitability of the experimental system for detecting the respective diterpene alcohol (Supplemental Fig. S2).

To further investigate the catalytic properties of IpTPS19, for which no activity was observed in *N. benthamiana*, the gene was cloned for heterologous expression in *Escherichia coli*. Crude protein extracts from overexpressing cells were used for in vitro enzymatic assays with the prenyl diphosphate substrates GPP, (*E*,*E*)-FPP, and (*E*,*E*,*E*)-GGPP; however, no terpenoid products were detected in single-enzyme assays (Supplemental Fig. S3, Supplemental Fig. S4). Given its phylogenetic placement within the TPS-e clade of diterpene synthases, IpTPS19 activity was then evaluated in coupled assays with the previously described poplar class II diterpene synthase PtTPS17 [37], which converts (*E*,*E*,*E*)-GGPP to *ent*-copalyl diphosphate (*ent*-CPP). In this two-enzyme system, IpTPS19 catalyzed the formation of *ent*-kaurene, presumably using *ent*-CPP as substrate, consistent with class I diterpene synthase activity (Supplemental Fig. S5). This catalytic behavior parallels that of its closest poplar orthologue, PtTPS19, and supports a role for IpTPS19 in *ent*-kaurene biosynthesis rather than in the production of geranyllinalool or other diterpenes (Supplemental Fig. S5).

### IpCyp82L45 catalyzes homoterpene formation in vitro and in planta

**Fig. 5:**
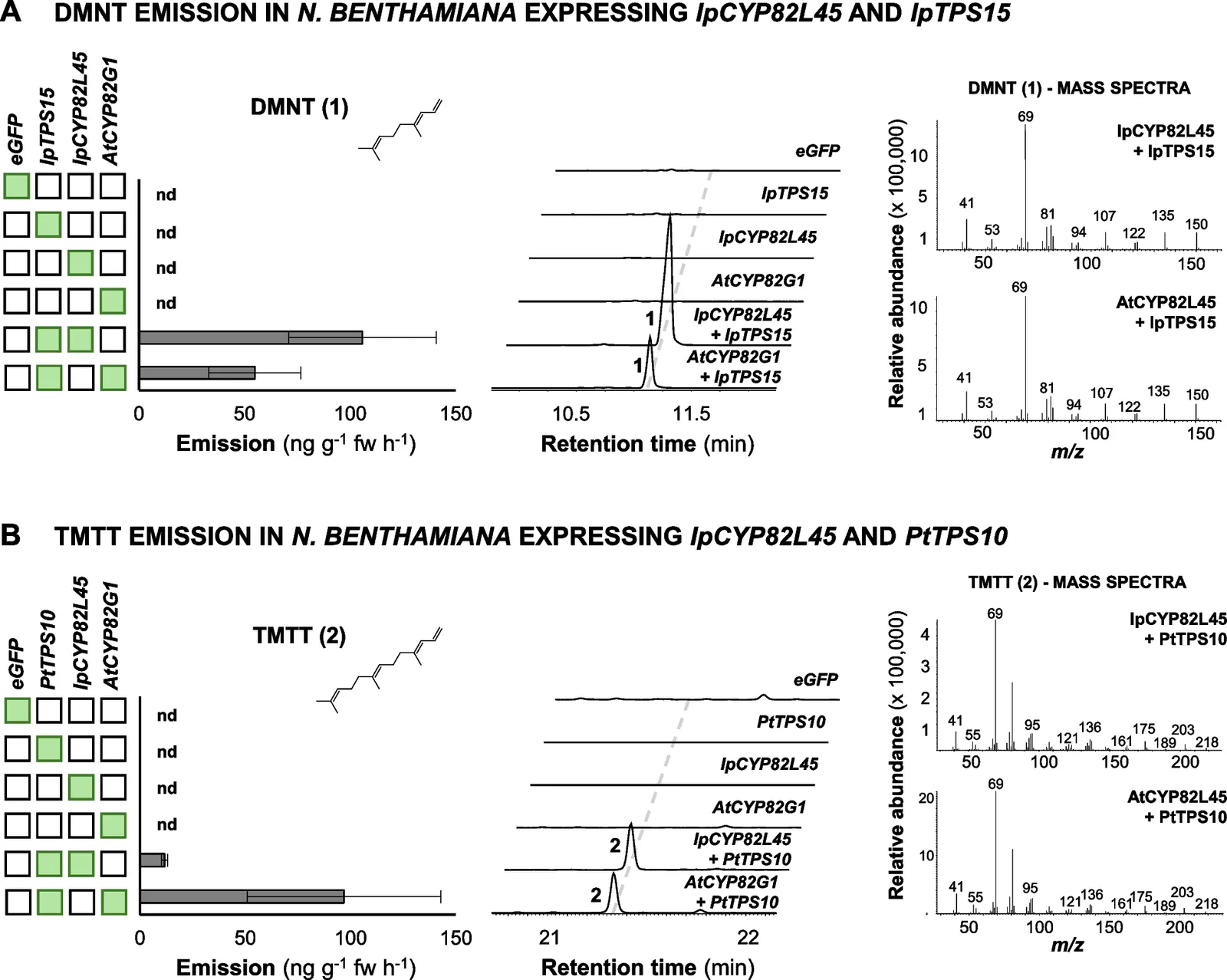
Volatile emissions from *Nicotiana benthamiana* plants transiently co-expressing *Idesia polycarpa CYP82L45* with *IpTPS15* or *PtTPS10*. Volatile emissions, representative GC chromatograms, and mass spectra are shown for *IpCYP82L45* in combination with *IpTPS15* (**A**) or *PtTPS10* (**B**). Data with the co-expression of the previously described *AtCYP82G1* are shown as a positive control and for product verification. Plants were infiltrated with *Agrobacterium tumefaciens* carrying *35S:eGFP*, *35S:IpTPS15*, *35S:PtTPS10*, *35S:IpCYP82L45*, and/or *35S:AtCYP82G1*. Volatiles were collected four days after infiltration over the course of 24 h using an active push–pull system and analyzed by gas chromatography–mass spectrometry (GC–MS) and quantified using gas chromatography–flame ionization detection (GC–FID). Data are presented as means ± SE (*n* = 3 biological replicates). DMNT, (3*E*)−4,8-dimethyl-1,3,7-nonatriene; fw, fresh weight; nd, not detected; TMTT, (*E*,*E*)−4,8,12-trimethyl-1,3,7,11-tridecatetraene. 1, DMNT; 2, TMTT. Compounds were identified by comparison with mass spectral databases and products formed by the positive control AtCYP82G1

To determine the role of IpCYP82L45 in homoterpene biosynthesis in *I. polycarpa*, we investigated the functional activity of this P450 candidate both *in planta* and in vitro. For *in planta* analysis, *IpCYP82L45* was transiently overexpressed in *N. benthamiana*, and volatile emissions were analyzed. Overexpression of *IpCYP82L45* alone did not result in detectable homoterpene or other volatile emissions (Fig. 5). However, co-expression of *IpCYP82L45* with the identified (*E*)-nerolidol synthase *IpTPS15* led to the emission of DMNT (Fig. 5A), and co-expression with the poplar geranyllinalool synthase *PtTPS10* resulted in emission of the C_16_ homoterpene TMTT (Fig. 5B). As a positive control, co-expression of the previously characterized *A*. *thaliana CYP82G1* with *IpTPS15* or *PtTPS10* likewise produced DMNT or TMTT, respectively, confirming the enzymatic activity of IpCYP82L45 in these heterologous combinations (Fig. 5).

To further validate its activity, IpCYP82L45 was heterologously expressed in yeast, and microsomal fractions were incubated with potential terpene alcohol substrates in the presence of NADPH. In these assays, IpCYP82L45 efficiently converted (*E*)-nerolidol to DMNT, consistent with the homoterpene formation observed *in planta*, whereas no activity was detected with (*Z*)-nerolidol (Supplemental Fig. S6A/B). When supplied with (*E*,*E*)-geranyllinalool, IpCYP82L45 showed no enzymatic activity (Supplemental Fig. S6C). Parallel assays using AtCYP82G1 confirmed the expected conversion of (*E*)-nerolidol and validated the assay conditions, while also showing no activity with (*Z*)-nerolidol or (*E*,*E*)-geranyllinalool (Supplemental Fig. S6). Together, these results show that IpCYP82L45 catalyzes homoterpene formation both in vitro and *in planta*, and support a role for this enzyme in herbivore-induced DMNT biosynthesis in *I. polycarpa*.

## Discussion

Herbivore-induced plant volatiles (HIPVs) are key components of plant defense that function in both direct resistance and indirect defense through the recruitment of natural enemies. Although HIPV biosynthesis and function have been extensively studied in herbaceous model systems, comparatively little is known about their evolutionary conservation and diversification in woody species. In this study, we characterized the volatile response of the non-model tree *Idesia polycarpa* following feeding by a generalist caterpillar to assess the conservation and lineage specificity of volatile-mediated defenses within the Salicaceae. Herbivory elicited a pronounced emission of volatiles, with the homoterpene DMNT as the dominant compound. Biochemical and functional analyses identified a sesquiterpene synthase and a cytochrome P450 monooxygenase that together mediate DMNT biosynthesis both in vitro and *in planta*. Our findings therefore extend previous work on herbaceous model systems and demonstrate that the conserved enzymatic logic of DMNT biosynthesis also operates in a woody member of the Salicaceae.

### IpCYP82L45 functions as a strong candidate enzyme underlying herbivore-induced DMNT biosynthesis in *I. polycarpa* leaves

Terpenes constitute the largest and most structurally diverse class of plant specialized metabolites and are typically classified according to the length of their carbon backbone as monoterpenes (C_10_), sesquiterpenes (C_15_), and diterpenes (C_20_) [8, 21, 22, 46]. In addition to these regular isoprenoid products, plants also produce irregular terpenes, such as the homoterpenes DMNT (C_11_) and TMTT (C_16_), which are generated via oxidative cleavage of higher-order terpene alcohol precursors [9, 29–31]. The biosynthetic sequence leading to DMNT formation in aboveground plant tissues is widely conserved across the plant kingdom [9]. It typically involves the formation of the sesquiterpene alcohol (*E*)-nerolidol by a TPS-g class I sesquiterpene synthase, followed by oxidative cleavage catalyzed by a cytochrome P450 monooxygenase, yielding the characteristic C_11_ homoterpene backbone [9, 12, 29–31]. Comparative studies indicate, however, that distinct P450 families have been recruited in a lineage-specific manner to catalyze this reaction. In several dicot species, including *Arabidopsis*, cotton, citrus, and, as shown here, *Idesia polycarpa*, members of the CYP82 family mediate DMNT formation (Fig. 5; [29–31]). In contrast, in the monocot maize, a CYP92 family member, CYP92C5, catalyzes the corresponding oxidative degradation step in DMNT formation [12]. Both the CYP82 and CYP92 families represent versatile cytochrome P450 families that participate in diverse branches of plant specialized metabolism. Members of the CYP82 family have also been implicated in benzylisoquinoline alkaloid biosynthesis, where they catalyze oxidative tailoring reactions that contribute to structural diversification of alkaloid scaffolds [47, 48]. Likewise, the CYP92 family exhibits considerable functional diversity and has been associated with benzoxazinoid biosynthesis, sesamol formation, and salicylic acid metabolism [49–51]. These examples underscore both the conservation of the overall biosynthetic logic underlying homoterpene formation and the evolutionary flexibility with which distinct P450 families have been recruited to catalyze analogous oxidative reactions, at least in aboveground tissues. Tissue-specific variation further illustrates the metabolic plasticity of plant specialized metabolism. In *Arabidopsis* roots, for instance, DMNT formation has been shown to proceed via breakdown of the triterpene diol arabidiol by CYP705A1, demonstrating that distinct precursor molecules can converge on homoterpene production depending on tissue context [18].

In addition to DMNT, its C_16_ analog TMTT is formed through oxidative cleavage of the C_20_ diterpene alcohol (*E*,*E*)-geranyllinalool [9]. TMTT was not detected in the herbivore-induced volatile bouquet of *I. polycarpa* leaves (Fig. 1; Supplemental Table S1). However, our heterologous overexpression experiments combining the poplar geranyllinalool synthase PtTPS10 [36] with IpCYP82L45 demonstrated that the enzyme is capable of forming TMTT, indicating that substrate availability rather than enzyme activity likely limits TMTT production in *I. polycarpa* (Fig. 5). Comparative transcriptome analysis did not identify a diterpene synthase candidate capable of producing geranyllinalool in *I. polycarpa*, but instead revealed a diterpene synthase generating *ent*-kaurene, a key intermediate in gibberellin biosynthesis (Supplemental Fig. S5; [52]). These results suggest that the diterpene biosynthetic network in *I. polycarpa* leaves may be primarily specified for growth-related metabolites rather than herbivore-induced volatile diterpenes. Comprehensive genome mining and functional characterization of TPS-e/f and TPS-c subfamily members will be required to determine the full complement and diversification of diterpene synthases in this species.

### Phytohormone-dependent regulation of herbivore-induced volatile biosynthesis

Jasmonates, particularly jasmonic acid (JA) and its bioactive conjugate JA-Ile, are central regulators of herbivore-induced metabolic reprogramming and play a key role in coordinating plant defense responses, including the production of herbivore-induced plant volatiles (HIPVs) [20]. JA signaling has been shown to activate transcriptional reprogramming of defense-related genes, including those involved in specialized metabolite biosynthesis, thereby linking herbivore perception to volatile emission [19, 20]. This regulatory role has been demonstrated in several plant species, including members of the Salicaceae family such as *Populus nigra*, *Populus trichocarpa*, and *Salix purpurea*, where herbivore attack induces both jasmonate accumulation and HIPV emission [19, 36, 53, 54]. In the present study, we likewise observed a significantly induced accumulation of JA and JA-conjugates following herbivory in *I. polycarpa*, indicating activation of jasmonate signaling in this system (Fig. 2). However, these data reflect a correlation between jasmonate levels and herbivore-induced responses and do not provide direct evidence for a causal role of JA in regulating DMNT biosynthesis. While JA- and salicylic acid (SA)-mediated pathways often interact antagonistically, more complex or even synergistic interactions have also been reported in woody species such as *Populus nigra* [55]. In contrast, *I. polycarpa* showed no clear antagonistic or synergistic SA response, but rather a relatively high constitutive SA level (Fig. 2). Overall, our results suggest that jasmonate accumulation might be associated with herbivore-induced volatile formation in *I. polycarpa* leaves, but functional studies will be required to resolve its regulatory role in DMNT biosynthesis in this woody species.

### Ecological role of DMNT in herbivore-induced plant volatile responses

Volatile organic compounds such as DMNT play important roles in mediating plant responses to their environment, influencing both herbivores and neighboring organisms [2, 56]. In *I. polycarpa*, herbivory by *Lymantria dispar* triggered high emission of the homoterpene DMNT. Previously conducted behavioral assays indicated that DMNT alone does not significantly alter *L. dispar* caterpillar behavior, suggesting that repellence is unlikely the primary defensive mechanism in this system [57]. Nevertheless, DMNT may still contribute to direct defense by impairing for instance herbivore performance, as reported for *Plutella xylostella*, where DMNT exposure could reduce larval growth, affect midgut integrity and pathogen susceptibility [17]. Such effects may be further enhanced when DMNT acts in combination with other volatiles in the induced blend. Indeed, the volatile bouquet emitted by *I. polycarpa* comprises a complex mixture of terpenoids and other compounds, which may act synergistically to influence herbivore responses (Fig. 1, Supplemental Table S1), potentially explaining why individual compounds sometimes fail to elicit detectable behavioral effects in isolation.

Beyond direct defense, DMNT may also serve additional ecological roles. In other plant systems, such as sweet potato (*Ipomoea batatas*), herbivory-induced DMNT mediates plant–plant signaling by inducing defense responses in neighboring individuals [13]. Thus, it is tempting to speculate that in long-lived trees, similar volatile signals could coordinate defense responses between spatially separated branches within the canopy. Taken together, these observations suggest that DMNT emission in *I. polycarpa* may contribute to multiple ecological processes, including direct defense and potential multitrophic or interplant signaling, although these roles remain to be experimentally confirmed.

## Conclusion

Herbivory by *Lymantria dispar* strongly induces mono- and sesquiterpene emissions in *I. polycarpa* leaves, with DMNT as the predominant compound. Transcriptomic and biochemical analyses indicate that DMNT formation depends on the coordinated activity of the TPS-g enzyme IpTPS15 and the cytochrome P450 IpCYP82L45, whereas the absence of a geranyllinalool synthase likely explains the lack of TMTT emission. Collectively, these results establish the molecular basis of homoterpene biosynthesis in a non-model woody species and demonstrate that CYP82-dependent DMNT formation is conserved in a woody member of the Salicaceae. Overall, our findings provide a molecular framework for future studies on the ecological and evolutionary significance of herbivore-induced plant volatiles in long-lived plants.

## Supplementary Information

Supplementary Material 1. Figure S1. Amino acid sequence comparison of the putative Idesia polycarpa cytochrome P450 monooxygenase (IpCYP82L45) and the Populus trichocarpa cytochrome P450 moonoxygenases (PtCYP82L1, PtCYP82L2) with the characterized CYP82G1 from Arabidopsis thaliana (At) [23]. The amino acid alignment was computed using the MUSCLE algorithm implemented in MEGA11 [46] and visualized with the program BioEdit (http://www.mbio.ncsu.edu/bioedit/bioedit.html). Black boxes mark conserved residues and gray boxes mark similar amino acids. The representative conserved domains among the P450s have been indicated as follows: HBD, heme-binding domain; PD, PERF domain; PRD, proline-rich domain. SRS1-6 indicate substrate recognition sites as predicted by [23]. Figure S2: (E,E)-geranyllinalool accumulation in Nicotiana benthamiana leaves transiently expressing Idesia polycarpa TPS19 or Populus trichocarpa TPS10. Displayed are diterpene alcohol accumulation (A) and representative GC chromatograms (B) of N. benthamiana leaf extracts expressing IpTPS19 or PtTPS10. Data with the expression of the previously described PtTPS10 [29] are shown as a positive control. Plants were infiltrated with Agrobacterium tumefaciens carrying 35S:eGFP, 35S:IpTPS19, or 35S:PtTPS10. Compounds were extracted with n-hexane from fresh N. benthamiana leaf material and analyzed by gas chromatography–mass spectrometry (GC–MS) and quantified using gas chromatography–flame ionization detection (GC–FID). Data are presented as means ± SE (n = 3 biological replicates). fw, fresh weight; nd, not detected. 1, (E,E)-geranyllinalool*. Compounds marked with ‘*’ were identified by comparison of their retention time and mass spectrum with those of authentic standards. Figure S3: In vitro enzymatic characterization of Idesia polycarpa TPS19. The enzyme was heterologously expressed in Escherichia coli, partially purified, and incubated with the substrates geranyl diphosphate (GPP), (E,E)-farnesyl diphosphate ((E,E)-FPP), or (E,E,E)-geranylgeranyl diphosphate ((E,E,E)-GGPP) and the cofactor Mg2+. Mono- and sequiterpene products were collected with a solid-phase microextraction (SPME) fiber and subsequently analyzed by GC-MS. Diterpene enzyme assays were overlaid with n-hexane, and the resulting organic phase was analyzed by GC-MS. Figure S4: In vitro assay controls using empty vector and no-protein reactions. Enzyme assays were conducted with protein raw extract from E. coli expressing an empty vector (EV) (A), or without enzyme (B). Empty vector (EV) was heterologously expressed in Escherichia coli, partially purified, and incubated with the substrates geranyl diphosphate (GPP), (E,E)-farnesyl diphosphate ((E,E)-FPP) or (E,E,E)-geranylgeranyl diphosphate ((E,E,E)-GGPP) and the cofactor Mg2+. Mono- and sequiterpene products were collected with a solid-phase microextraction (SPME) fiber and subsequently analyzed by GC-MS. Diterpene enzyme assays were overlaid with n-hexane, and the resulting organic phase was analyzed by GC-MS. Figure S5: In vitro enzymatic characterization of Idesia polycarpa TPS19 and Populus trichocarpa TPS19 in combination with P. trichocarpa TPS17. Enzyme activity, including representative GC chromatograms (A) and mass spectra (B), are shown for product formation by the combinations PtTPS17 with IpTPS19 and PtTPS17 with PtTPS19. The enzymes were heterologously expressed in Escherichia coli and partially purified. Enzymatic activity was determined for the previously characterized PtTPS17 [30] with the substrate (E,E,E)-geranylgeranyl diphosphate ((E,E,E)-GGPP), as well as for PtTPS17 in combination with the previously characterized PtTPS19 [30] or the IpTPS19 candidate. Diterpene enzyme assays were overlaid with n-hexane, and the resulting organic phase was analyzed by GC–MS. 1, ent-kaurene. Compounds were identified by comparison with mass spectral databases and with products formed by the positive control PtTPS19. Figure S6: In vitro enzymatic activity of Idesia polycarpa CYP82L45. The enzyme was heterologously expressed in Saccharomyces cerevisiae (WAT11) and microsomes were purified. Microsomal fractions were used in enzymatic assays with the substrates (E)-nerolidol (A), (Z)-nerolidol (B), or (E,E)-geranyllinalool (C) and the cofactor NADPH. AtCYP82G1 was used as a positive control, as previously described for DMNT and TMTT formation [23]. Products were collected with a solid-phase microextraction (SPME) fiber and analyzed by GC–MS. 1, DMNT; 2, TMTT. Compounds were identified by comparison with mass spectral databases and by comparison with the products formed by the positive control AtCYP82G1. DMNT, (3E)-4,8-dimethyl-1,3,7-nonatriene; EIC, extracted ion count. Figure S7: Controls for the in vitro enzymatic activity of IpCYP82L45. IpCYP82L45 was heterologously expressed in Saccharomyces cerevisiae (WAT11), and microsomes were purified. Enzyme assays were performed under the following control conditions: (A) without NADPH, (B) without substrate, (C) without microsomes, and (D) with heat-inactivated microsomes. Products were collected with a solid-phase microextraction (SPME) fiber and analyzed by GC–MS. EIC, extracted ion count.

Supplementary Material 2. Table S1. Volatiles emitted by undamaged (ctr) and gypsy moth caterpillar (Lymantria dispar, herb) damaged Japanese orange cherry (Idesia polycarpa) leaves. Volatile compounds were collected using a push–pull system and subsequently analyzed and quantified by gas chromatography–mass spectrometry (GC–MS) and gas chromatography-flame ionization detection (GC–FID), respectively. Mean (ng g-1 FW h-1) ± SE (n = 6 biological replicates). Compounds marked with ‘*’ were identified by comparison of retention time and mass spectrum to those of authentic standards. Compound marked with ‘#’ by comparison of retention time and mass spectrum to those of the previously described P450 enzyme AtCYP82G1 [23]. Other compounds were identified by database comparisons. Differences between treatments were analyzed by a Student's t test (ST) or Mann-Whitney Rank Sum Test (MW) (* *P* < 0.05; ** *P* < 0.01; *** *P* < 0.001). DMNT, (E)-4,8-Dimethyl-1,3,7-nonatriene; fw, fresh weight; nd, not determined. Table S2. In silico analysis of functional domains and subcellular localization of identified Japanese orange cherry (Idesia polycarpa) terpene synthase (TPS) candidates. Predicted nucleotide (nt) and amino acid (aa), terpene synthase type, conserved functional domains and the subcellular localization based on the analyses by TargetP 2.0 [32] are given. CTP, Chloroplast transfer peptide; DiTPS, diterpene synthase, MTS, monoterpene synthase; STS, sesquiterpene synthase. Table S3. Gene expression fold changes of candidate genes. Fold changes in Japanese orange cherry (Idesia polycarpa) leaves following herbivory by gypsy moth (Lymantria dispar) caterpillar compared with untreated leaves. P-values are given and are assessed by Student‘s t-test. Table S4. Oligonucleotides used for amplification of full-length (fl) and truncated (tr) terpene synthases (TPS) and cytochrome P450 monooxygenases (CYP) from Idesia polycarpa (Ip), Populus trichocarpa (Pt), and Arabidopsis thaliana (At). fwd, forward; rev, reverse.

Supplementary Material 3.

## Data Availability

Availability of data and materials All supporting data are included as additional files. Constructs described in this work and datasets analyzed during the current study are available from the corresponding author upon request. Sequence data for *IpTPS3* (PZ213723), *IpTPS15* (PZ213724), *IpTPS19* (PZ213722), and *IpCYP82L45* (PZ213725) can be found in the NCBI GenBank (https://www.ncbi.nlm.nih.gov/genbank/) under the corresponding identifiers. The RNA sequencing data generated in this study have been deposited in the NCBI Sequence Read Archive (SRA) under BioProject accession number PRJNA1473663. Associated BioSample accession numbers are SAMN60567041–SAMN60567046. Individual SRA run accession numbers are SRR38967400–SRR38967405.

## Abbreviations

LC–MS/MS: Liquid chromatography-tandem mass spectrometry
GC-MS: Gas chromatography-mass spectrometry
